# How does brief motivational intervention work? A mediation analysis

**DOI:** 10.1186/1940-0640-7-S1-A25

**Published:** 2012-10-09

**Authors:** Jacques Gaume, Nicolas Bertholet, Mohamed Faouzi, Gerhard Gmel, Jean-Bernard Daeppen

**Affiliations:** 1 Center for Alcohol Treatment, Department of Medicine and Public Health, Lausanne University, Lausanne, Switzerland

## 

Little is known about exactly which elements of alcohol brief motivational intervention (BMI) make it work. A causal chain between therapist motivational interviewing behaviors, subsequent client change talk (CT), and actual behavior change has been postulated. Other researchers have proposed the notion of a working alliance between counselor and client to explain better outcomes. However, these links have never been tested within a single mediation framework. We investigated the articulation of counselor behaviors, CT, working alliance, and six-month alcohol use outcomes (drinks per week at follow-up adjusted for drinks per week at baseline) during BMI with young men using regression analyses. There were no direct links from counselor skills to alcohol outcomes. Some counselors skills predicted more CT (MI-consistent behaviors frequency [p = 0.02] and MI spirit rating [p = 0.04]) as did working alliance (p < 0.001). However, CT did not predict the alcohol outcome. One subdimension of CT, ability/desire/need to change (ADN), did predict outcome (p = 0.04). None of the tested counselor skills predicted this dimension, but working alliance did (p = 0.03), giving a first significant model (better working alliance > more ADN > better outcomes). Adding a step in this model, we showed that the percent of MI-consistent behaviors and the MI spirit, empathy, and acceptance ratings predicted better working alliance scores (all p < 0.01). As the final step, we will calculate the mediated effects in these models. We found that MI skills and behaviors predicted a better working alliance, which, in turn, predicted more ADN change talk, which, in turn, predicted better alcohol use outcome. This gives support to previous findings but also highlights the importance of working alliance. Such findings might give precious clues for clinicians by giving an indication of the most determinant elements in BMI.

